# How does psychosocial safety climate cross-level influence work engagement and job burnout: the roles of organization-based self-esteem and psychological detachment

**DOI:** 10.1186/s12912-024-01935-8

**Published:** 2024-06-06

**Authors:** Tongshuang Yuan, Hui Ren, Xin Yin, Leilei Liang, Junsong Fei, Xiaoying Liu, Chengbin Zheng, Huimin Wang, Jiaying Gao, Songli Mei, Hongyan Li

**Affiliations:** 1 School of Public Health, Jilin University, No. 1163 Xinmin Street, Changchun, Jilin 130021 China; 2 Department of Nursing, The First Hospital of Jilin University, Changchun, Jilin 130021 China; 3 The First Hospital of Jilin University, No. 71 Xinmin Street, Changchun, Jilin 130021 China

**Keywords:** Psychosocial safety climate, Work engagement, Job burnout, Nurses, Cross-level model

## Abstract

**Background:**

Existing researches on nurses’ work engagement and job burnout have mostly stayed at the individual level, and limited researches test the cross-level effects of psychosocial safety climate (PSC). The study aimed to explore the cross-level mediating effect of organization-based self-esteem (OBSE) and the moderating effect of psychological detachment between the relationship of PSC and work engagement and job burnout in nurses.

**Methods:**

The cross-sectional study was conducted during November to December 2022 at a tertiary hospital in a northeastern province of China. Data was collected from 1832 nurses through an online questionnaire. Correlation analyses and hierarchical linear modeling were used to test study hypotheses.

**Results:**

The results showed that PSC was positively associated with work engagement, and negatively associated with job burnout. OBSE mediated the effect of PSC on work engagement, as well as job burnout. Additionally, psychological detachment played a moderating role between PSC and work engagement, but no moderating effect was found between PSC and job burnout.

**Conclusions:**

PSC at the organizational level increases work engagement and reduces job burnout by stimulating nurses’ high levels of OBSE. Psychological detachment, as a situational factor, enhances the positive influence of PSC on work engagement. The implementation of measures to improve the PSC levels of the organization, and the levels of OBSE and psychological detachment among nurses could help to promote their good work performance.

**Supplementary Information:**

The online version contains supplementary material available at 10.1186/s12912-024-01935-8.

## Introduction

As the largest group of health care professionals, nurses are essential participants in achieving high quality care and positive health outcomes in patients [1]. Safeguarding the quality of care and increasing the nurses’ willingness to stay in the workforce are important to support people’s health and well-being, and to safeguard the stability and growth of health care systems. Work engagement has grown in prevalence over the past 25 years as an important predictor of job performance and outcomes [2]. Work engagement is a state of individuals’ continuous, positive emotional activation related to work, characterized by three aspects: vitality, dedication and concentration [3]. Previous studies results point out that work engagement is closely related to nurses’ psychological, physical, behavioral and organizational outcomes, such as reducing psychological disorders, reducing work errors, improving nursing services, reducing their tendency of leaving, and increasing the economic benefits of hospitals [4].

Simultaneously, increased work demands and inadequate work resources cause nurses to be negatively affected by work-related stressors for long duration and increase the incidence of job burnout [5]. A meta-analysis showed that the global incidence of nurses’ burnout is high and on an upward trend, and that the COVID-19 pandemic has led to an increase in the upward trend [6]. Job burnout refers to a syndrome involving emotional exhaustion, dehumanization and diminished accomplishment under prolonged work stress [7]. Higher levels of burnout could lead to various mental and physical health problems in nurses such as anxiety, depression, insomnia, headaches, and chest pain. Meanwhile, when nurses suffer from high level of burnout, patient safety and quality of care decline and the intention of leaving increases [8, 9]. Given the significant impacts of work engagement and job burnout on individual and organizational outcomes, it is critical to explore the factors that promote work engagement and reduce job burnout among nurses in healthcare settings.

**Fig. 1:**
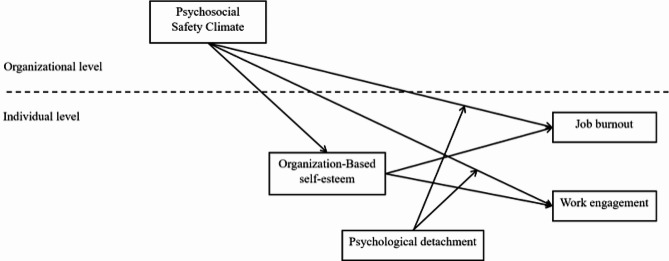
Theoretical model

However, existing researches on nurses’ work engagement and job burnout have mostly stayed at the individual level. Kacey Keyko et al. proposed the Nursing Job Demands-Resources for nursing practice, which emphasized the vital influence of organizational-level factors on nurses’ physical and psychological health, job performance and nursing outcomes [10]. Psychosocial safety climate (PSC), as a key resource at the organizational level, has attracted a lot of attentions. PSC refers to policies, practices and procedures established by the organization regarding employees’ psychological health and safety in the course of their work [11]. PSC has been shown to be strongly associated with employees’ physical and mental health, work attitudes, and job performance, and is more strongly correlated with psychological health outcomes such as burnout than other organizational measures [12, 13]. Given the challenges and stresses nurses face in their work environments, our study aimed to better understand how to promote work engagement and prevent job burnout, and to use the PSC as an important organizational resource to achieve this goal in order to further consider promoting positive work outcomes by focusing on the mental health and safety of nurses. However, limited researches test the cross-level effects of PSC on work engagement and job burnout, and there are also unknown factors in the mediating mechanisms of the above relationship, such as from the perspective of individuals’ resources, i.e., organization-based self-esteem (OBSE). Furthermore, the influence of PSC on work engagement and job burnout may have boundary conditions. It is necessary to explore in depth the moderating effect of individual characteristics (psychological detachment) in the PSC’s influence mechanism from the perspective of human-environmental interaction. Based on this, the present study constructed a cross-level model of PSC at the organizational level affecting nurses’ work engagement and job burnout, used OBSE as a mediator to further clarify its mechanism, and focused on the moderating effect of psychological detachment in the above relationships. In order to provide theoretical guidance for the implementation of measures to promote PSC in organizations to enhance nurses’ mental health and work performance. In summary, the model for this study is shown in Fig. 1.

### PSC and work engagement, as well as job burnout

PSC was included in the Job Demands-Resources (JD-R) model as an antecedent variable that affected job characteristics and it was considered as an active organizational variable acting on health-impairment process and motivational process [14]. In a high PSC context, people’s mental health goals are valued, supported, committed and prioritized by senior managers [15, 16]. Correspondingly, multiple resources are provided to employees, thus facilitating them access to more conditional, individual, and workplace resources. Individuals can use these resources to regulate their own emotional responses, thereby alleviating the negative impact of various stressors in the work environment. Consequently, the prevalence of job burnout and mental health problems will be reduced, and individuals’ work engagement would be increased. In addition, according to social exchange theory (SET) [17], when an organization cares for members’ mental health and well-being through rational allocation of resources and priority management, members would fully perceive the organization’s support. Based on the reciprocity’s principle, individuals will have a sense of obligation to reciprocate. In exchange, they will reward the organization with more positive attitudes and behaviors, which could elevate organizational commitment and work engagement. Taken together, we propose the hypotheses that:

H1a. PSC at the organizational level is positively related to work engagement.

H1b. PSC at the organizational level is negatively related to job burnout.

### PSC and OBSE

Self-esteem, as a component of the self-concept, reflects the perception of the self and encompasses both evaluation and cognitive orientation. People with high self-esteem generally have more positive views and feelings about themselves and have a higher sense of “self-liking” [18]. OBSE reflects individual’s self-esteem in the specific area of work and organizational environment, and is the self-judgement on one’s own value, ability, and importance in the organization [19]. OBSE is highly situational in nature, and it has been shown that that performance feedback, organizational climate, managerial roles and related behaviors can all affect OBSE [20]. When the health and well-being of employees is a priority for the organization, that is, in the case of a high level of PSC, managers usually keep work demands within limits and increase work resources to reduce the negative impact of factors such as work stress on employees [12]. As a result, nurses perceive that they are valued by the organization and may make judgments that their work behaviors and outcomes are affirmed by the organization. Such information is incorporated into their self-perceptions, allowing them to form their own positive evaluations of the organization as having value and competence. Therefore, we hypothesize that:

H2: PSC at organizational level is positively related to OBSE.

### Mediating effect of OBSE

Combined with H2, when nurses perceive high levels of PSC, their OBSE levels increase. When nurses perceive that their mental health and safety is important to management and the organization, their positive emotions and motivation increase. And PSC plays a role in reducing work demands and improving work resources [12], as a result, nurses perceive that the organization values and recognizes them and tend to positively evaluate their value in the organization, and their OBSE levels are enhanced. When people actively evaluate their values in the organization, the organizational identity becomes the content of the constructed self-concept and an important component of the self-concept system [21]. The consistency theory assumes that people are motivated to achieve conjunctions that are consistent with their self-concept [22]. Nurses with high OBSE levels maintain consistency in self-evaluation through a series of positive behaviors and responses, which in turn could maintain or even increase them self-esteem levels in the organization and achieve the maintenance of positive self-concept [23, 24]. Based on this, nurses tend to be more willing to put in work and demonstrate more positive efforts and enthusiasm to strengthen their self-perception and value. Conversely, nurses would align their self-perception by exhibiting pessimistic work-related attitudes and behaviors. Eventually, nurses’ work commitment would be undermined and the risk of job burnout would increase. In addition, according to Conservation of Resource Theory (COR) [24], OBSE, as an individual resource, can motivate individuals to work and thus increase their levels of work engagement. The higher levels of OBSE, the higher quality sense of meaning, security, and access they experience in their work role engagement, which in turn shows higher levels of work engagement and lower levels of job burnout. And, high levels of OBSE would help nurses build and access more resources that would further contribute to their positive psychological experiences at work and reduce job burnout levels. Accordingly, we propose the hypotheses that:

H3a. OBSE mediates the relationship between PSC and work engagement.

H3b. OBSE mediates the relationship between PSC and job burnout.

### Moderating effect of psychological detachment

Psychological detachment refers to a state in which people is temporally, spatially, and psychologically detached from work after work time, is not disturbed by work-related matters, and stops thinking about work-related issues [25]. Work engagement is a positive emotional state that lasts for a long time and requires the consumption physical and psychological resources. The effort-recovery model revealed that people responded to work demands through effortful behaviors which depleted their resources, so that short breaks allow their physical and mental systems to recover properly. Psychological detachment has been found to reduce energy expenditure and allow people to acquire new resources and perspectives form other activities, so they can return to work with better state [26]. Conversely, nurses who cannot detach from work and continue to devote time and energy to work-related tasks after working hours. This will lead to further depletion of personal resources and elevated emotional exhaustion, and harder to adequate recovery [25]. On this basis, we propose that,

H4a. Psychological detachment moderates the relationship between PSC and work engagement.

H4b. Psychological detachment moderates the relationship between PSC and job burnout.

## Methods

### Study design

The study data was collected from a tertiary hospital in a northeastern province of China during November to December 2022, collected through cluster sampling procedures. 1,832 nurses from 17 departments (such as obstetrics and gynecology, oncology, pediatrics, and intensive care unit) participated in the survey.

### Participants and procedure

First, we contacted the head of the nursing department and relevant administrators to illustrate the purpose and procedures of the investigation. After obtaining their consent, electronic questionnaires were created and distributed through the ‘Questionnaire Star platform’. The platform enables online survey for data collection and download. Once the online questionnaire was created, the head of nursing department forwarded the information and the QR code of the investigation to chief nurses via the WeChat group, who then distributed the questionnaire to the WeChat groups of nurses in each department. Nurses were fully aware of study purpose and procedures and chose to participate voluntarily. The survey was conducted anonymously to fully protect the privacy of the participants. Online informed consent was obtained from all participants.

A total of 1868 questionnaires were received in the current study, and 1832 questionnaires were collected ultimately after deleting those with missing key variables, obvious regular answers or repeated answers, with a valid recovery rate of 98.07%. The survey collected more than half of the hospital’s nurse population and the basic demographic characteristics of the nurses surveyed were similar to those of the total population. The average age of participants was 35.11 (SD = 6.10) years old. The study was carried out in accordance with the Helsinki Declaration as revised 1989, and the study protocol was approved by the Institutional Review Board of the School of Public Health, Jilin University.

### Measurements

#### PSC

PSC was assessed by the 12-item psychosocial safety climate scale (PSC-12), which contains four subscales: management commitment, organizational communication, management priority, and organizational participation [27]. The Chinese version PSC-12 had good reliability and validity [28]. Each item was rated on a 5-point Likert scale from 1 (strongly disagree) to 5 (strongly agree). The total score was the sum of four subscales, with higher score values indicating higher levels of PSC. The Cronbach’s α coefficient in this study was 0.97.

#### OBSE

The Organization-Based Self-Esteem (OBSE) Scale developed by Pierce was used to measure the level of self-esteem at work [29]. An example item was “I am valuable around here”. The total four items were rated on a 5-point Likert scale, from 1 (strongly disagree) to 5 (strongly agree). The OBSE scale was proved to be a reliable and valid instrument [30]. The Cronbach’s α coefficient in this study was 0.88.

#### Work engagement

The 3-item short version scale developed by Christian et al. was utilized to measure work engagement [31]. Three items were as follows: “I was enthusiastic in my job today” (emotional engagement), “I was absorbed by my job today” (cognitive engagement), and “I exerted my full effort on my job today” (physical engagement). The answers to each item were rated on a 5-point Likert scale varying from 1 (strongly disagree) to 5 (strongly agree). The Cronbach’s α coefficient in this study was 0.89.

#### Job burnout

Job burnout was measured by the 10-item burnout subscale from the Professional Quality of Life (ProQOL) Scale developed by Stamm [32]. An example question was “I have happy thoughts and feelings about the people I help and how I can help them”. Items were rated on a 5-point Likert scale ranging from 1 (no) to 5 (always). The scale has shown good validity and reliability in previous research [33]. The Cronbach’s α coefficient in this study was 0.71.

#### Psychological detachment

Four items associating with psychological detachment derived from the recovery experience questionnaire were used to assess the psychological detachment [34]. The scale was widely used to measure the degree of psychological detachment [35]. Each item was responded with a 5-point Likert scale, ranging from1 (strongly disagree) to 5 (strongly agree). The Cronbach’s α coefficient in this study was 0.85.

#### Control variables

Demographic information, including gender (1 = “male”; 2 = “female”), age (1 = “≤30”; 2 = “31–40”; 3 = “>40”), education level (1 = “junior college or below”; 2 = “bachelor degree”; 3 = “master degree or above”), and monthly income (yuan) (1 = “≤6000”; 2 = “6001–8000”; 3 = “8001-10,000”; 4 = “>10,000”), and work-related variables, including technical title (1 = “none”; 2 = “nurse”; 3 = “senior nurse”; 4 = “supervisor nurse”; 5 = “co-chief nurse”; 6 = “chief nurse”), working years (1 = “≤4”; 2 = “5–9”; 3 = “10–14”; 4 = “≥15”), working time (hours)/week (1 = “≤40”; 2 = “41–45”; 3 = “>45”), and night shift (times)/week (1 = “0”; 2 = “1–2”; 3 = “3–4”; 4 = “≥5”) were measured as control variables. This is because these variables have been shown to potentially influence the hypothesized relationships in this study [36, 37]. Therefore, the inclusion of these variables helps to control for external factors that may have an impact on the results, thus allowing for a more accurate examination of the relationship between the study variables.

### Statistical analysis

In this study, the data analyses and hypotheses testing were conducted using the SPSS 24.0, Hierarchical Liner Modeling 6.08 and R 4.1.0. First, descriptive statistics and Pearson correlations were used to analyze the characteristics of the sample and the relationship between all study variables. Second, considering the study variables involve both individual level and organizational level, hierarchical linear modeling was used for hypothesis testing. Hierarchical linear modeling is a linear statistical analysis method for multi-layer nested structured data, capable of processing data from different levels simultaneously. In the present study, the level 2 represented data at the organizational level and the level 1 represented data at the individual level. The study used R-Mediation to calculate the 95% confidence interval (CI) of the mediating effect [38]. Statistical significance was defined as a two-tailed *p*-value smaller than 0.05.

## Results

### Common method bias test

The results showed that six factors with eigenvalues greater than 1 were extracted. The variance explained by the first factor was 39.45%, which was below the threshold of 50% [39]. The result indicated no serious common method bias in the measurements.

### Results of descriptive statistics and correlations

Table 1 displays the means, standard deviation, and correlation coefficients of the studied variables. The results showed that all the control variables were significantly associated with work engagement and job burnout (*p*<0.05). PSC was significantly and positively correlated with OBSE and work engagement (*r* = 0.499, 0.512, *p*<0.001), and negatively correlated with job burnout (*r*=-0.477, *p*<0.001). OBSE was significantly and positively correlated with work engagement (*r* = 0.812, *p*<0.001), and negatively correlated with job burnout (*r*=-0.566, *p*<0.001). Psychological detachment was significantly and positively correlated with work engagement (*r* = 0.092, *p*<0.001), and negatively correlated with job burnout (*r*=-0.081, *p*<0.01).

**Table 1:** Descriptive statistics and Pearson correlation matrix of the study variables

Variables	M	SD	1	2	3	4	5	6	7	8	9	10	11	12
Individual level variables(*n* = 1832)														
1.Gender	1.92	0.27	1											
2.Age	1.94	0.58	0.152^***^	1										
3.Educational level	1.99	0.32	0.118^***^	0.131^***^	1									
4.Monthly income	3.17	0.90	0.021	0.233^***^	0.180^***^	1								
5.Technical title	3.37	0.83	0.127^***^	0.509^***^	0.310^***^	0.296^***^	1							
6.Working years	2.64	0.94	0.128^***^	0.812^***^	0.137^***^	0.247^***^	0.521^***^	1						
7.Working time	2.22	0.70	0.014	0.028	0.047^*^	0.049^*^	0.052^*^	0.018	1					
8.Night shift	2.24	0.84	-0.093^***^	-0.288^***^	-0.081^***^	-0.042	-0.199^***^	-0.245^***^	-0.001	1				
9.OBSE	3.78	0.74	0.042	0.104^***^	0.069^**^	0.082^***^	0.109^***^	0.111^***^	-0.091^***^	-0.148^***^	1			
10.Work engagement	3.77	0.78	0.052^*^	0.120^***^	0.058^*^	0.063^**^	0.096^***^	0.126^***^	-0.074^**^	-0.121^***^	0.812^***^	1		
11.Job burnout	2.47	0.59	-0.114^***^	-0.086^**^	-0.065^**^	-0.086^***^	-0.107^***^	-0.082^***^	0.127^***^	0.187^***^	-0.566^***^	-0.609^***^	1	
12.PD	2.92	0.89	-0.027	-0.035	-0.038	-0.017	-0.076^**^	-0.049^*^	-0.060^*^	0.018	0.101^***^	0.092^***^	-0.081^**^	1
Organizational level variables(*n* = 17)														
PSC	3.49	0.93	0.065^**^	-0.017	0.035	-0.033	0.025	-0.023	-0.134^***^	-0.087^***^	0.499^***^	0.512^***^	-0.477^***^	0.137^***^

Notes: ^*^p < 0.05, ^**^p < 0.01, ^***^p < 0.001, OBSE: organization-based self-esteem, PSC: psychosocial safety climate, PD: psychological detachment

### Aggregation statistics

PSC, an organizational-level variable derived from multiple evaluations on nurses at the individual level, should be aggregated to the organizational level in the actual analysis. R_wg_ and two intraclass correlation coefficients (ICC) were calculated, and used to determine the aggregation’s reasonableness of individual-level data to organization-level data. R_wg_ was used to confirm whether the data of each department has high intraclass consistency [40], and in general, R_wg_ value should be greater than 0.70 [41]. ICC(1) examines whether there is sufficient between-group variation in the variable, with a larger value representing greater variation between groups, and ICC(1) is usually required to be greater than 0.059 [42]. ICC(2) indicates the organizational variables’ reliability after aggregation of individual variables to the organizational level, and ICC(2) should be greater than 0.70 [42]. In this study, the mean and median R_wg_ of PSC were 0.78 and 0.76, respectively, indicating a high intraclass consistency of the survey data. ICC(1) and ICC(2) were 0.06 and 0.87, respectively, indicating that the survey data had good intraclass stability and variability, thus supporting the effective aggregation of PSC from the individual level to the organizational level.

### Hypotheses testing

The direct effects of PSC on OBSE, work engagement, and job burnout at the individual level all involved cross-level relationships, so three null models needed to be construct. ICCs were 0.066, 0.104 and 0.082 of OBSE, work engagement and job burnout, respectively, in accordance with the judgment criteria of ICC (1) greater than 0.059 (see supplementary material table S1). The between-group differences in OBSE (F = 5.018, *p* < 0.001), work engagement (F = 6.108, *p* < 0. 001), and job burnout (F = 6.422, *p* < 0.001) were significant, thus requiring a cross-level analysis to examine the between-group differences in the above variables.

The study used hierarchical linear modeling to verify the cross-level mediating effect, and the variables other than the dependent variables were group-mean centered. The results are shown in Table 2. After controlling for demographic information and work-related variables, there was a positive relationship between PSC and work engagement (M3, γ = 0.828, *p*<0.001). Thus, H1a was supported. PSC was positively associated with OBSE (M1, γ = 0.630, *p*<0.001). Thus, H2 was supported. OBSE was positively correlated with work engagement (M2, γ = 0.858, *p*<0.01). OBSE had a significant positive effect on work engagement when it entered M4 (M4, γ = 0.845, *p* < 0.001), while the coefficient of significant effect of PSC on work engagement decreased from 0.828 to 0.259, with OBSE partially mediated the relationship between PSC and work engagement. Furthermore, the result of the Monte Carlo method (5,000 replications) showed that the coefficient of indirect effect was significant (indirect effect = 0.491, 95%CI=[0.443, 0.623]). The 95%CI did not contain zero, which confirmed that OBSE played a mediating role in the association between PSC and work engagement. Thus, H3a was supported.

**Table 2:** Mediating results of OBSE on the relationship between PSC and work engagement, and PSC and job burnout

	Parameter	OBSE	Work engagement			Job burnout		
		Model 1	Model 2	Model 3	Mode 4	Model 5	Model 6	Model 7
	Fixed effects							
Level 1	Intercept(γ_00_)	3.901^***^	3.782^***^	3.870^***^	3.788^***^	2.594^***^	2.544^***^	2.590^***^
	Gender	-0.069	0.017	-0.044	0.015	-0.145^**^	-0.113^*^	-0.145^***^
	Age	0.020	0.012	0.030	0.021	0.019	0.015	0.015
	Educational level	0.081^*^	0.026	0.082	0.023	-0.016	-0.058	-0.015
	Monthly income	0.039^*^	-0.013	0.018	-0.012	-0.027	-0.047^**^	-0.030^*^
	Technical title	0.027	-0.012	0.004	-0.018	-0.021	-0.033	-0.021^*^
	Working years	0.018	0.037	0.058	0.035^*^	0.009	-0.005	0.008
	Working time	-0.084^***^	-0.011	-0.076^**^	-0.006	0.067^**^	0.103^***^	0.069^***^
	Night shift	-0.119^**^	0.013	-0.086^**^	0.013	0.068^***^	0.123^**^	0.067^***^
	OBSE		0.858^**^		0.845^***^	-0.449^***^		-0.443^***^
Level 2	PSC	0.630^***^		0.828^***^	0.259^***^		-0.518^***^	-0.259^***^
	σ^2^(within-group variation)	0.461	0.503	0.196	0.196	0.222	0.313	0.222
	τ_00_(between-group variation)	0.003	0.080	0.002	0.001	0.005	0.005	0.002

Note: ^*^P < 0.05, ^**^P < 0.01, ^***^P < 0.001, OBSE: organization-based self-esteem, PSC: psychosocial safety climate

The same procedure was adopted to verify the mediating role of OBSE in the relationship between PSC and job burnout. There was a negative relationship between PSC and job burnout (M6, γ=-0.518, *p*<0.001). Thus, H1b was supported. OBSE was negatively correlated with job burnout (M5, γ=-0.449, *p*<0.001). OBSE had a significant negative effect on burnout when it entered M7 (M7, γ=-0.443, *p*<0.001), while the coefficient of significant effect of PSC on job burnout decreased from − 0.518 to -0.259, with OBSE partially mediated the effect between PSC and job burnout. Further, the Monte Carlo method (5,000 replications) results showed that coefficient of indirect effect was significant (indirect effect − 0.279, 95%CI=[-0.326, -0.232]). The 95%CI did not contain zero, which confirmed that OBSE mediated the association between PSC and job burnout. Thus, H3b was supported.

**Fig. 2:**
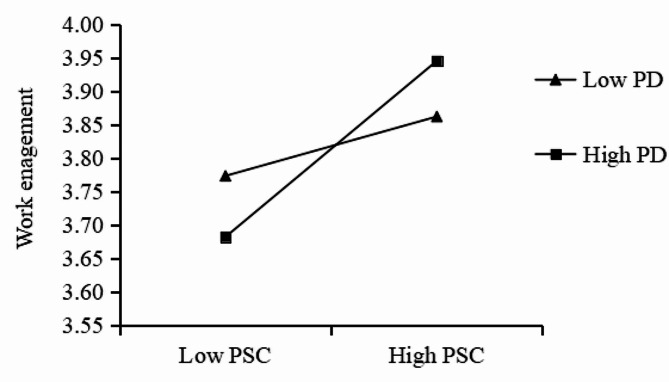
The interactive effect of PSC and PD on work engagement. (Note: PSC: psychosocial safety climate, PD: psychological detachment)

In this study, when validating the cross-level interaction effect, the data of organizational-level PSC (as the independent variable) was grand-mean centered, and the psychological detachment at the individual level (as the moderator) was group-mean centered [43]. The moderating effects of psychological detachment in the relationship between PSC and work engagement, as well as job burnout are shown in Table 3. The results indicated that the interaction between PSC and psychological detachment was positively and significantly related to work engagement (γ = 0.158, *p*<0.05), but the interaction was insignificantly related to job burnout (γ=-0.135, *p*>0.05). To better visualize the moderating role of psychological detachment, the simple slope test as recommended by Aiken et al. was utilized [44]. Figure 2 presents a plot of the interaction effect between psychological detachment (M ± 1SD) and PSC at different levels. As shown in Fig. 2, the relationship between PSC and work engagement was stronger for higher level psychological detachment than lower level.

**Table 3:** Moderating results of PD on the relationship between PSC and work engagement, and PSC and job burnout

	Parameter	Work engagement	Job burnout
	Fixed effects		
Level 1	Intercept(γ_00_)	3.799^***^	2.570^***^
	Gender	0.013	-0.140^***^
	Age	0.022	0.017
	Educational level	0.018	-0.013
	Monthly income	-0.013	-0.029^*^
	Technical title	-0.016	-0.021^*^
	Working years	0.034	0.007
	Working time	-0.008	0.069^**^
	Night shift	0.016^*^	0.063^***^
	OBSE	0.846^***^	-0.446^***^
Level 2	PSC	0.283^**^	-0.222^***^
	psychological detachment	-0.012	0.029
	PSC*PD	0.158^*^	-0.135
	σ^2^(within-group variation)	0.196	0.222
	τ_00_(between-group variation)	0.000	0.008

Note: ^*^P < 0.05, ^**^P < 0.01, ^***^P < 0.001, OBSE: organization-based self-esteem, PSC: psychosocial safety climate, PD: psychological detachment

## Discussion

To the knowledge of the authors, this is the first study to reveal the influence of PSC on work engagement and job burnout in Chinese nurses and to examine the mediating role of OBSE and the moderating role of psychological detachment based on a large sample. The results showed that the effect of PSC differs from person to person and enriched the situational analysis of PSC’s mechanism. And relative to the moderators incorporated in previous studies (such as individual fixed characteristics and external resources), psychological detachment has developable, actionable characteristics and significant value. The findings provide theoretical support and guidance for the organization and manager to improve nurses’ work engagement and reduce job burnout.

First, this study found that PSC had a positive effect on work engagement and a negative effect on job burnout, which is consistent with previous research findings [16]. At a high level of PSC, the organization’s senior management takes mental health and safety seriously and endeavors to ensure job requirements are reasonable. By creating good working conditions, nurses are protected from mental health problems and their work engagement can be improved [45]. Meanwhile, PSC acts as ‘resource caravan passageways’ to promote motivational process of the JD-R model through the organization at all levels of management or the organization’s resources, thus producing a spiral gain effect of resources [16]. Nurses’ existing resources are protected and more new resources are developed, generating a resource caravan phenomenon [46]. Hence, in a high-level PSC scenario, nurses are more likely to have access to work-related psychological resources to reduce the negative effects of work-related stress and demands. Moreover, when nurses perceive higher level of PSC, it means that they perceive that the organization values and supports their mental health. Nurses are more likely to develop a ‘perceived obligation’ to repay the organization with more positive work attitudes and work behaviors. This finding is also supported by SET.

Second, this study confirmed that OBSE mediated the relationship between PSC and work engagement, as well as job burnout. Specifically, PSC positively predicted OBSE, and OBSE positively predicted work engagement, and negatively predicted job burnout. Similar findings pointed to a mediating role of OBSE between environmental resources and job crafting behaviors [47]. The positive organizational climate has a motivating effect on the spirit of individuals and stimulates their intrinsic motivation. Therefore, high PSC increases nurses’ perception of their own values in the organization, which in turn raises their OBSE. Based on the self-consistency theory, nurses maintain and strengthen their self-perceptions by developing more positive work attitudes and behaviors [48]. This finding is also supported by COR theory. OBSE reflects nurses’ sense of self-worth as members of the organization, which acts as a valuable workplace resource and can stimulates nurses’ loyalty and commitment to the organization. When nurses feel respected and recognized by the organization, they are motivated to participate in their work and devote more time and experience to their tasks. And they are better able to face challenges at work and face problems with a more positive attitude, which reduces burnout levels [49].

Finally, psychological detachment significantly moderated the relationship between PSC and work engagement. Previous studies have similar confirmations. For instance, Yu et al. revealed that psychological detachment moderated the relationship between stressors and innovative work behavior [50]. This may be because that when nurses achieve a state of physical and mental separation from work during non-working time, they would recover and acquire new resources from leisure activities. When they return to work again, they could deal with their work in a better condition, which promotes the positive impact of PSC on work engagement. However, psychological detachment did not have a significant moderating effect in the association between PSC and job burnout. This may be due to the fact that high levels of PSC provide nurses with abundant resources, and even nurses with lower levels of psychological detachment still have access to relatively adequate resources to maintain their good emotional state. This suggests that a lack of psychological detachment does not necessarily increase job burnout. Lack of psychological detachment only becomes a problem when work resources are inadequate, because it means that individuals will be continually and negatively affected by work-related stressors. Notably, previous studies suggested that high level of psychological detachment could lead to more severe burnout and depression when work resources were insufficient [51]. These findings emphasize that the psychological detachment does not always produce positive outcomes and that work resources still are a key factor in reducing burnout and the risk of mental health problems.

### Implication

In summary, the study expands the pathway mechanisms and boundary conditions of PSC affecting nurses’ work engagement and job burnout. These findings emphasize the significant value of interventions at the organizational level. Managers should consider nurse psychological safety as one of the key organizational goals. The attention and behavioral practices of managers to nurses’ mental health could be enhanced through leadership styles’ training and development. Furthermore, the findings indicate that OBSE may act as a potential intervention target to increase work engagement and reduce job burnout through the implementation of programs that promote OBSE. Finally, the importance of appropriate detachment from work should be given full attention. Accordingly, organizations and managers should reduce communication with nurses during non-working time and rationalize work demands accordingly to allow and support nurses to detach during that time. At the meanwhile, nurses should be offered psychological detachment interventions, guidance, and training to enable them to better detach from their busy and stressful work. This would contribute to positive work performance and quality of care for nurses.

### Limitation

There were some limitations to consider in this study. Firstly, the cross-sectional design limited the ability to draw causal conclusions, and made it difficult to examine the dynamic processes by which PSC influenced work engagement and job burnout. The present study tried to control variables that may have an impact on the research hypotheses in order to minimize the influence of confounding factors on the results. To address the issue of common method bias arising from the cross-sectional design, this study collected nurses with different characteristics (including age, monthly income, technical title, working years, etc.), and the hierarchical linear modeling addressed common method bias in the individual-level data to some extent by modeling organization-level PSC and estimating between-team effects [52]. However, given the limitations of the cross-sectional study design, future studies may try to add consideration of the temporal dimension based on a multilevel design, while using methods such as scenario-based experiments. And it can be combined with qualitative research in order to explore in depth the mechanisms and reasons behind the relationships between the research variables. Secondly, we recruited participants from only one hospital, and the context of the present study should be considered when summarizing our findings. Despite the relatively large sample size collected, the generalizability of the findings to other populations and countries should be carefully assessed. Future studies are encouraged to be conducted in a wider range of populations as well as other cultures to increase the external validity of the findings. Thirdly, the data were measured by self-report approach, which may have introduced common method bias [39]. Future research could take the form of collecting data at multiple points in time. Also, where possible, objective indicators are used to verify the veracity of the data, for example, by using field or behavioral observations, assessments by others, and so on.

## Conclusion

By constructing a cross-level theoretical model, this study provided a better understanding of “through what mechanisms” and “in what contexts” PSC at organizational level influences work engagement and job burnout among nurses. The study results showed that PSC was positively associated with work engagement and negatively associated with job burnout through OBSE. Our analysis also showed that high level of psychological detachment enhanced the positive effect of PSC on work engagement, but not the negative effect of PSC on job burnout. From the perspective of PSC, the study identified the influential mechanisms and situational characteristic of nurses’ work engagement and job burnout. The findings provide important insights in the management and practice for hospital managers and organizations to promote positive work engagement and psychological well-being among nurses.

### Electronic supplementary material

Below is the link to the electronic supplementary material.

Supplementary Material 1

## Data Availability

The data used or analyzed during the current study are available from the corresponding author upon reasonable request.
