# Supplementary material for: How does psychosocial safety climate cross-level influence work engagement and job burnout: the roles of organization-based self-esteem and psychological detachment
Source: BMC Nurs. 2024 Jun 6;23:389. doi: 10.1186/s12912-024-01935-8 (PMC11154979; doi:10.1186/s12912-024-01935-8)
Supplement: Supplementary file 1 — Supplementary Material 1 [file 12912_2024_1935_MOESM1_ESM.doc]

Table S1 Results of null models

| Index | Organization-based self-esteem | Work engagement | Job burnout |
| --- | --- | --- | --- |
| σ^2^(within-group variation) | 0.488 | 0.542 | 0.335 |
| τ_00_(between-group variation) | 0.035 | 0.063 | 0.030 |
| ICC(1) | 0.066 | 0.104 | 0.082 |
